# Cost analysis of acute care resource utilization among individuals with sickle cell disease in a middle-income country

**DOI:** 10.1186/s12913-021-07461-6

**Published:** 2022-01-08

**Authors:** Clarisse Lobo, Patricia Moura, Delaine Fidlarczyk, Jane Duran, Roberto Barbosa, Thais Oliveira, Emilia Matos do Nascimento, Nickhill Bhakta, Jane S. Hankins

**Affiliations:** 1 Instituto Estadual de Hematologia do Rio de Janeiro HEMORIO, Rio de Janeiro, RJ Brazil; 2 Fundação Centro Universitário Estadual da Zona Oeste UEZO, Rio de Janeiro, Brazil; 3 Department of Global Pediatric Medicine, St Jude Children’s Research Hospital, 262 Danny Thomas Place, TN 38105 Memphis, USA; 4 Department of Hematology, St. Jude Children’s Research Hospital, TN 38105 Memphis, USA

**Keywords:** Sickle cell anemia, Pain, Hospitalization, Admission, Financial analysis, Emergency room, Emergency department, Micro-costing, Acute complications, Hydroxycarbamide, Medication possession ratio

## Abstract

**Background:**

The costs associated with the treatment of sickle cell disease (SCD) are understudied in low and middle-income countries (LMIC). We evaluated the cost of treating SCD-related acute complications and the potential cost-savings of hydroxyurea in a specialized hematology center in Brazil.

**Methods:**

The costs (US dollars) of emergency department (ED) and hospitalizations from SCD-related complications between 01.01.2018 and 06.30.2018 were ascertained using absorption and micro-costing approaches. The reasons for acute hospital visits were grouped as: 1) vaso-occlusive (VOC) pain, 2) infection, 3) anemia exacerbation, and 4) chronic organ damage complications. Hydroxyurea adherence was estimated by medication possession ratio (MPR) during the study period.

**Results:**

In total, 1144 patients, median age 17 years (range 0–70), 903 (78.9%) with HbSS/HbSβ^0^-thalassemia, 441 (38.5%) prescribed hydroxyurea, visited the ED, of whom 381 (33%) were admitted. VOC accounted for 64% of all ED visits and 60% of all admissions. Anemia exacerbation was the most expensive reason for ED visit ($321.87/visit), while chronic organ damage carried the highest admission cost ($2176.40/visit). Compared with other genotypes, individuals with HbSS/HbSβ^0^-thalassemia were admitted more often (79% versus 21%, *p* < 0.0001), and their admission costs were higher ($1677.18 versus $1224.47/visit, *p* = 0.0001). Antibiotics and analgesics accounted for 43% and 42% of the total ED costs, respectively, while housing accounted for 46% of the total admission costs. Costs of ED visits not resulting in admissions were lower among HbSS/HbSβ^0^-thalassemia individuals with hydroxyurea MPR ≥65% compared with visits by patients with MPR <65% ($98.16/visit versus $182.46/visit, *p* = 0.0007). No difference in admission costs were observed relative to hydroxyurea use.

**Discussion:**

In a LMIC hematology-specialized center, VOCs accounted for most acute visits from patients with SCD, but costs were highest due to anemia exacerbation. Analgesics, antibiotics, and housing drove most expenses. Hydroxyurea may reduce ED costs among individuals with HbSS/HbSβ^0^-thalassemia but is dependent on adherence level.

**Supplementary Information:**

The online version contains supplementary material available at 10.1186/s12913-021-07461-6.

## Introduction

Sickle cell disease (SCD) is a pleiotropic genetic disorder of the hemoglobin with a chronic course of progressive end-organ dysfunction superimposed by recurrent episodes of acute vaso-occlusion (VOC) leading to frequent acute care resource utilization [i.e., visits to the emergency department (ED) and hospitalizations]. Additionally, due to the loss of splenic function early in life, individuals with SCD are at high risk of infections from encapsulated organisms, prompting additional acute hospital visits in the event of fever, which is considered an emergency in SCD [1].

Childhood survival among patients with SCD has improved in the last decades. In high-income countries (HIC), greater than 95% of children with SCD survive to age 18 years [2]. In low-and middle- income countries (LMIC), such as Brazil, improvements in survival have also been observed [3, 4]. Initially recognized as a juvenile disease, SCD is now increasing in prevalence among adults in Brazil as more children and adolescents survive [5]. As a result, SCD has become a chronic medical disease in Brazil, characterized by a pattern of recurrent episodes of acute events and progressive organ dysfunction as seen in HIC, [6] collectively leading to increased acute care utilization and financial burden on the health care system [7]. Therefore, SCD represents a public health challenge for many countries, especially LMIC, where resources are scarcer and decisions about resource allocation are based on competing priorities.

Although there are some data regarding the costs associated with SCD in Europe and the United States, [8, 9] such data are not yet available for LMICs. The aim of this study was to evaluate the costs associated with acute complications of SCD at a large publicly-funded hematology center in Brazil, over a 6-month period. We hypothesized that there is variability in cost relative to reason for hospital visit, age and exposure to hydroxyurea. Our objectives were to 1) estimate the costs associated with acute care treatment of children and adults with SCD, according to the reason for the hospital visit, and 2) explore the potential cost-savings of hydroxyurea therapy during acute care visits.

## Methods

### Study setting and participant selection

We included all patients (children and adults) with a diagnosis of SCD (of any genotype) treated at the public hematology referral center, HEMORIO, Rio de Janeiro, Brazil, between 01.01.2018 and 06.30.2018, and selected all those who experienced an acute care visit (ED visit) due to a SCD-related cause for inclusion in the acute care cost analysis. As per institutional policy, all patients admitted to the inpatient setting at HEMORIO must be seen and treated in the ED first, therefore, all hospital admissions are a subset of the ED visit encounters. HEMORIO is a State-funded tertiary hematology treatment center carrying for approximately 2000 children and 1300 adults with SCD. In use since 1998, HEMORIO’s clinical database extracts data from all the institution’s patients’ health records by queries made through the Microsoft SQL Server, using the development tool in Visual FoxPro 9 system. This system unites, in a single platform, the complete patient care management. This solution allows the integration of data and processes across different sectors of the hospital, providing centralized information management and quality control of patient care. The HEMORIO’s clinical database was queried to identify participants who met the study’s inclusion criteria. ED visits and hospitalizations (hospital admissions) that resulted from non-SCD related causes (e.g., trauma) or were not a result of an acute complication from SCD (e.g., routine lab visits, routine transfusions) were excluded from the cost analysis. A retrospective chart review of all clinical notes for each acute care visit was manually conducted to verify eligibility. All hydroxyurea prescriptions filled during the period of analysis were included. The treatment of acute SCD-related complications followed local standards of practice and guidelines set forth by the Brazilian Ministry of Health (Supplemental Table 1). The HEMORIO Ethics Committee approved the secondary use of existing data. All methods were carried out in accordance with relevant guidelines and regulations.

### Cost analysis

The ED and hospital admission costs (both separated and combined) were aggregated into fixed and variable categories for all patients over the period of the study. Fixed costs were generally identified using institutional budgets while variable costs were quantified using a microcosting approach [10]. Fixed costs included in the study were: personnel time, imaging and laboratory tests, administrative time (including Pharmacy costs for drug storage and dispensation), and specialty consulting fees (e.g., psychology, infectious diseases specialist) (Supplemental Table 2). For personnel costs, the required time for patient care was calculated by multiplying the time spent in direct patient care (i.e., the estimated effort % of each provider) by the annual salary of all staff with primary patient care responsibilities (e.g., physicians, nurses, physical therapists). All other fixed costs were determined based on a combination of staff time spent treating patients with SCD and HEMORIO salary hourly rate [11]. For variable costs, a microcosting approach, where all consumables were accounted for through primary data abstraction from the HEMORIO database, was used to estimate the cost of medications, laboratory tests, blood transfusions and sub-specialty consultations. Using a standardized process, all observed medical orders were abstracted from each encounter within each individual health record during the study period. The total cost for the ED and hospital admission visits were calculated by summing the line items for the fixed and variable costs.

Because all hospital admissions were subsets of ED visits, we classified an ED visit that did not result in an admission as a “treat and release ED visit”, or simply “ED visit”. If an ED encounter resulted in an admission, then the encounter was classified as an “ED visit converted into and admission visit”, henceforth simply referred to as an “admission visit”. The total cost of care per ED or admission visit was estimated by dividing the total fixed and variable cost for all patients seen in the ED or admitted by the total number of respective acute visits in the study period. ED costs are presented as cost per ED visit (per each event) and admission costs are presented as cost per each admission (per each event).

The cause of ED or hospital admission visit was verified by a physician and abstracted from the medical record during the retrospective chart review. After removing all non-SCD related encounters, all remaining ED and admission visits were grouped into 4 discharge diagnoses: 1) VOC defined by acute pain crisis, priapism or dactylitis; 2) Infection defined by fever, sepsis or acute chest syndrome (ACS); 3) Anemia exacerbation defined by acute hemolytic crisis, transient aplastic crisis, or acute splenic sequestration; 4) Chronic organ damage defined by brain (overt stroke), kidneys (chronic kidney disease), liver (chronic liver disease) dysfunction or failure.

Per-ED and per-admission visit costs were calculated for each discharge diagnosis group and dichotomized by age (≤18 or > 18 years) as previously done [12]. Due to the use of budget data in the cost assessment and to ensure that the data matched observed costs, a secondary validation was conducted by assessing outliers where total costs calculated were < 2.5% or > 97.5% of encounters in our cost analysis. Thirty charts were reviewed at random to determine the cause of each outlier. The reasons for each were catalogued and no systematic deviations between our reported and observed costs were identified. All costs are shown in 2018 US Dollars and calculated by converting from Brazilian Reais (R$) to US Dollars ($) using a conversion rate of $1 US Dollar = R$ 3,18 (as of June 30, 2018).

### Statistical considerations

Descriptive statistics are provided as total count and percentage for categorical variables and median and range for continuous variables. To ensure that the period of the analysis was representative of the acute care utilization pattern at HEMORIO, the Kruskal-Wallis test was used to verify the existence of seasonality between the periods. The period from January 2018 to July 2018 was compared with the previous semester (July 2017 to December 2017) and subsequent semester (July 2018 to December 2018). There were no differences in the number of ED or admission encounters per day when comparing the different periods (*p* = 0.71). Additionally, there were no differences in patient numbers with HbSS/HbSβ^0^-thalassemia (*p* = 0.56) or other sickle genotype (*p* = 0.13) per segment.

The one-sample proportions test with continuity correction was used to compare the clinical outcome differences (i.e., frequency of ED or admissions) across discharge diagnoses groups and the Wilcoxon rank sum test with continuity correction was used to compare time and cost differences across the groups. Total absolute and relative costs were stratified by discharge diagnosis and type of medication. Adherence to hydroxyurea was estimated by the Medication Possession Ratio (MPR) of hydroxyurea, defined as the ratio between the number of days of hydroxyurea possession up to the date of the ED visit and the total number of days from the beginning of the period of analysis to the date of the same ED visit [13]. A non-parametric regression tree model was built to analyze the association between the costs of allED visits with the following independent variables: age (< 18 or ≥ 18 years), hydroxyurea MPR, sex, sickle genotype (Hb SS or HbSB^0^thalassemia versus other genotypes), discharge diagnosis, and admission (yes or no). Analyzes were conducted using R software version 4.0.3 (The R Foundation).

## Results

### Sample characteristics

During the study period there were 3331 active patients with SCD at HEMORIO. In this period, a total of 2700 ED visits (with or without subsequent admission) occurred among 1144 unique patients with SCD. Twenty-nine (1.1%) visits were excluded because they were not due to an acute SCD-related diagnosis (Supplemental Table 3). Therefore, a total of 2671 ED visits (with or without subsequent admission) among 1144 unique patients were included in the analysis.

Of these 1144 patients, 579 (50.6%) were male and 565 (49.4%) were female, with a median age of 17 years (range 0 to 70 years); 600 (52.4%) were children (age ≤ 18 years) and 441 (38.5%) were prescribed hydroxyurea at the time of the ED visit (Table 1). Hydroxyurea was prescribed to 40.5% of the children and 36.4% of adults. The sickle genotype distribution of these 1144 patients was: 903 (78.9%) HbSS or HbSβ^0^-Thalassemia; 174 (15.2%) Hb SC; 48 (4.2%) with HbSβ^+^-Thalassemia; and 19 (1.7%) with HbSD. Among the 1144 patients who visited the ED during the period of the analysis, 381 (33.3%) were admitted at least once during the study period, and the remaining 763 (66.7%) patients were treated and released from the ED (Table 1).

**Table 1:** Participants’ characteristics

	Total	Patients seen in the ED and released	Patients seen in the ED and admitted
Number of unique patients (n,%)	1144 (100%)	763 (66.7%)	381 (33.3%)
Age in years (mean; sd)^a^	20.4 (14.8)	21.6 (14.9)	17.9 (14.3)
Sex (n,%)			
Male	579 (50.6%)	381 (33.3%)	198 (17.3%)
Female	565 (49.4%)	382 (33.4%)	183 (16.0%)
Sickle genotype (n,%)			
HbSS and HbSβ^0^-thalassemia	903 (78.9%)	596 (52.1%)	307 (26.8%)
HbSC and HbSβ^+^-thalassemia	241 (21.1%)	167 (14.6%)	74 (6.5%)
Hydroxyurea therapy at any point during the study period (n,%)	441 (38.5%)	285 (24.9%)	156 (13.6%)

^a^Age as of the beginning of the observation period

Of the 2671 ED visits included, 519 of them resulted in a hospital admission, therefore, 2152 ED visits were treat-and-release, and 519 were admissions. Eight (0.7%) patients died during either the ED (*n* = 3) visit or admission (*n* = 5). Aggregate patient-level characteristics are shown for these ED visits, stratified by their outcome of treat-and-release versus admission (Table 2).

**Table 2:** Characteristics of all ED visits stratified by visit outcome

	All ED visits	ED visits treat-and-release	ED visits resulting in admission
Total number of events	2671 (100%)	2152 (80.6%)	519 (19.4%)
Age (years) (mean; sd)^a^	22.6 (15.5)	23.2 (15.4)	19.8 (15.4)
Sex (n,%)			
Male	1464 (54.8%)	1188 (44.5%)	276 (10.3%)
Female	1207 (45.2%)	964 (36.1%)	243 (9.1%)
Sickle genotype			
HbSS and HbSβ^0^-thalassemia	2103 (78.7%)	1693 (63.3%)	410 (15.4%)
HbSC and HbSβ^+^-thalassemia	568 (21.3%)	459 (17.2%)	109 (4.1%)
Hydroxyurea therapy at the time of the ED visit (n, %)	1062 (39.8%)	843 (31.6%)	219 (8.2%)

^a^Age as of the ED encounter. *ED* emergency department

### Emergency department utilization

**Fig. 1:**
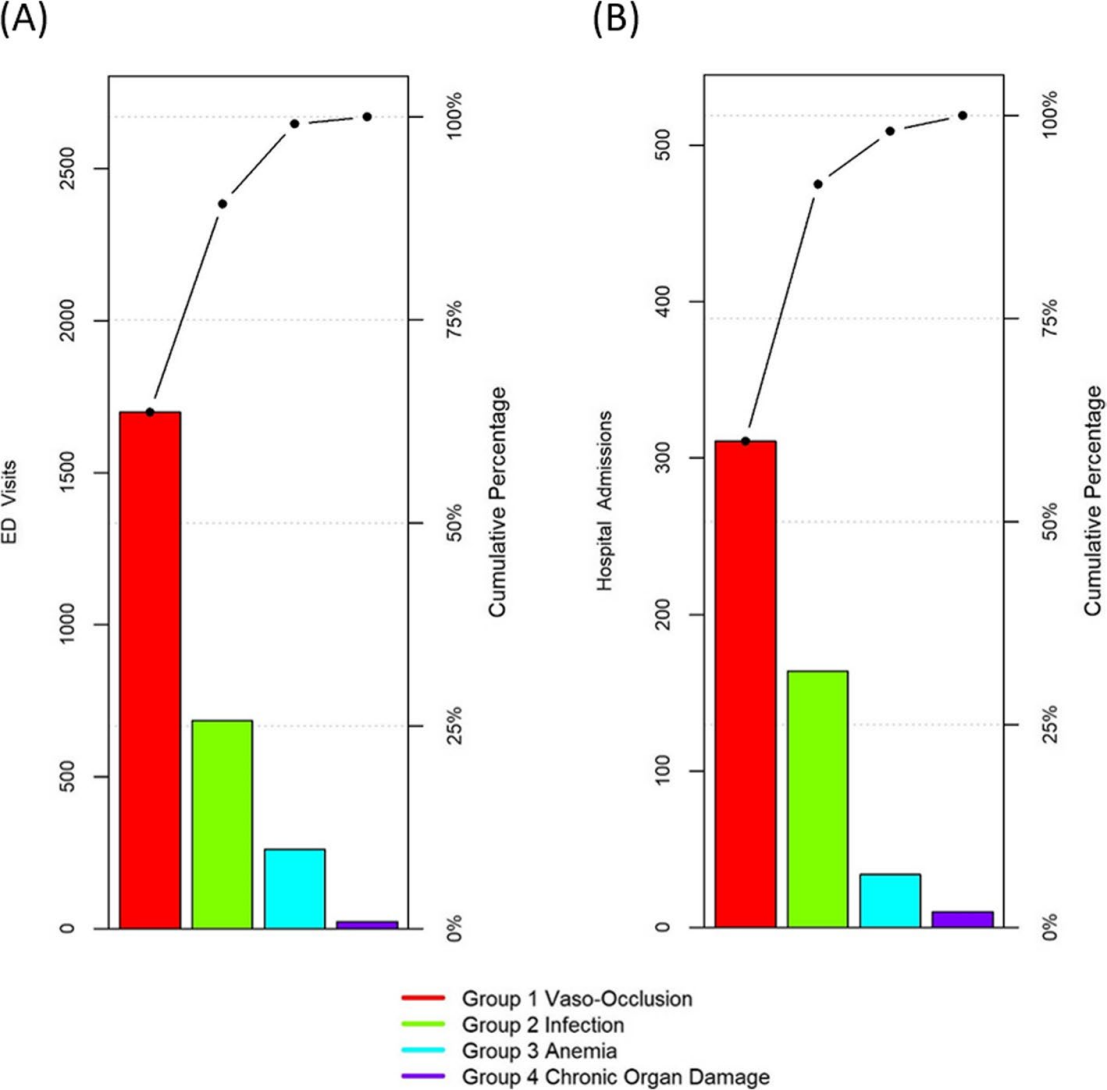
Pareto Chart of the Discharge diagnosis for Emergency Department (ED) and admissions. Most acute care visits were due to pain crisis or infection for both ED (**A**) and admission (**B**) visits

Most of the 2671 ED visits were due to VOC events, followed by infection, anemia exacerbation and organ damage (Fig. 1A). A total of 332 patients (29%) sought care in the ED > 2 times during the analysis period. Three deaths occurred in the ED: two due to acute meningitis, a 4-year-old girl with HbSC and a 7-year-old boy with HbSS and one due to hemorrhagic stroke in a 66-year-old HbSS male.

Overall, ED visits most commonly had individuals with HbSS or HbSβ^0^-thalassemia, males, and patients not treated with hydroxyurea relative to the prevalence of other sickle genotypes, females, and patients prescribed with hydroxyurea (Supplemental Fig. 1A, 1B, 1D).

VOC ED visits were mostly fulfilled by adults, whereas infection ED visits was mostly comprised of children (Supplemental Fig. 1C). Within all ED visits, there was a higher proportion of visits by patients who were not prescribed hydroxyurea than by those who had been prescribed this medication [703 (61.4%) versus 441 (38.5%), *p* < 0.0001). The median length of stay in the ED was highest for VOC (5.4 h) and anemia (5.2 h), and significantly higher than the other two discharge diagnoses (*p* = 0.027). Additionally, ED visits among patients with HbSS or HbSB^0^-thalassemia genotypes and adults had higher length of stay compared with that with other genotypes (*p* = 0.005) and children (*p* < 0.001), respectively (Supplemental Fig. 2A and C). There were no differences in length of stay in the ED according to sex or hydroxyurea prescribing.

### Costs associated with emergency department utilization

**Fig. 2:**
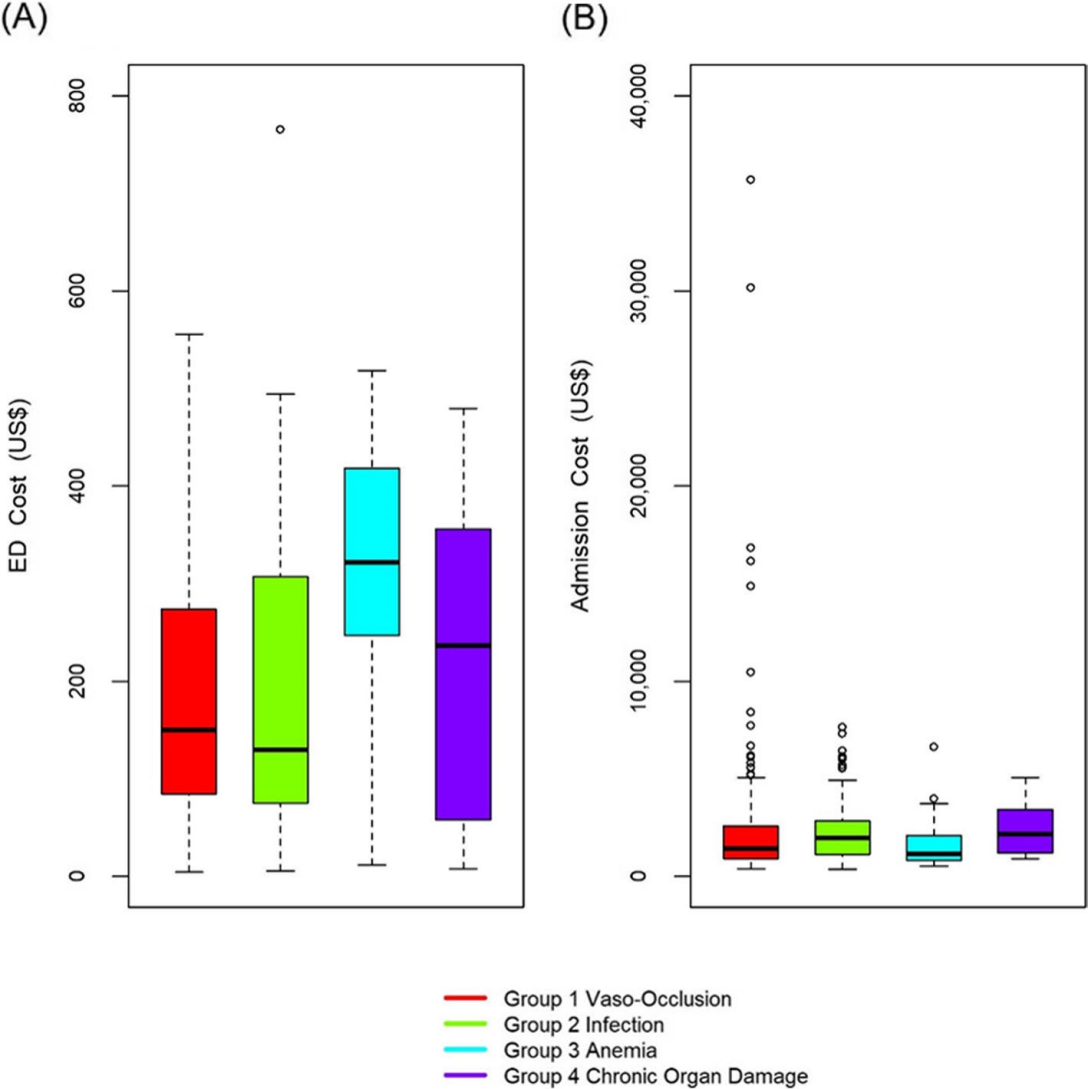
Mean costs for emergency department (ED) and the Admissions. Mean costs per each ED (**A**) and admission (**B**) visits stratified by discharge diagnosis group is shown. Among all the ED discharge diagnoses, anemia had the higher mean cost per event, while the highest cost per event among admissions was treatment of chronic organ damage

Anemia exacerbation was the most expensive cause for ED visit, with a total cost of $321.87 per visit, and significantly higher than the second most expensive cause for ED visit, organ damage ($236.40, *p* = 0.0026, Fig. 2A).

The cost of an ED visit due to acute anemia was highest among the subset of patients with HbSS or HbSβ^0^-thalassemia, median $330.62 per visit relative to visits with other genotypes (Supplemental Fig. 3A). There was no difference in ED cost relative to sex or age group. As an exploratory analysis, the costs for ED visits were analyzed excluding the value of the packed red blood cell concentrate, given its high cost. The median cost of ED visits due to VOC ($127.11) were slightly higher than the cost of those due to acute anemia, when excluding the use of blood products ($119.32, *p* = 0.02).

Finally, the cost of treatment during ED utilization was analyzed according to the prescription of hydroxyurea at the time of the visit. Median cost per visit to the ED were no different among individuals who were prescribed hydroxyurea or not ($148.09 versus $151.64 respectively, *p* = 0.136).

### Hospitalizations

A total of 519 admissions occurred among the 381 patients. Among the 519 hospitalizations, 311 (59.9%) were due to VOC, 164 (31.6%) due to infection, 34 (6.6%) due to anemia exacerbation and 10 (1.9%) due to chronic organ damage complications (Fig. 1B). Eighty-seven (22.8%) patients were hospitalized more than once, 67 of them due to VOC. Causes of death during hospitalizations included chronic organ failure (*n* = 1), sudden death during pain crisis (*n* = 1), ACS (*n* = 2) and sepsis (*n* = 1). Hospitalizations were mostly comprised of patients with HbSS or HbSβ^0^-thalassemia, males (except for admissions due to anemia exacerbation), and adults (except for admission due to infection) (Supplemental Fig. 1E, F,G).

Accounting all admissions, the proportion of children who received a blood transfusion was higher than that of adults: 145 (57.5%) versus 107 (42.5%), respectively (*p* = 0.0098). However, when considering the VOC discharge diagnosis only, there was no difference in the proportion of children (45%; *n* = 63) and adults (55%; *n* = 77) who received blood transfusions during a hospitalization (*p* = 0.2719). Similarly to ED visits, there was a significantly higher proportion of admissions by patients who were not prescribed hydroxyurea than by patients admitted and prescribed this medication (57.8% versus 42.2%, *p* = 0.0002, Supplemental Fig. 1H). Additionally, admissions due to infection also had higher proportion of patients not prescribed hydroxyurea compared with that of those prescribed this medication [109 (66.5%) versus 55 (33.5%), *p* < 0.0001, Supplemental Fig. 1H).

The overall median admission length of stay for the entire group was 3.9 days. The longest length of stay was due to chronic organ dysfunction (median 5.2 days), followed by infection (median 5.1 days) and VOC (median 5.7 days), however many outliers were observed. Overall length of stay was higher among children compared with that of adults 4.81 and 2.83 days, respectively, *p*-value < 0.0001. However, adults trended towards longer lengths of stay for infection relative to children (6.87 versus 4.93 days, respectively, *p* = 0.0541). The median length of stay for children with a VOC (4.83 days) was longer compared with adults (2.77 days) (*p* < 0.0001). The median length of stay was longer for individuals with HbSS or HbSβ^0^-thalassemia (4.26 days) than other genotypes (2.77 days) *p* = 0.0006. Patients prescribed hydroxyurea had longer lengths of stay (4.47 days) than those not prescribed it (3.77 days, *p* = 0.0086, Supplemental Figure, 2E, 2H). There was no difference in length of stay relative to age or sex (Supplemental Fig. 2G, F). Finally, although carrying low adherence values overall, among patients prescribed hydroxyurea, MPR was significantly higher among those not admitted compared with those admitted (38.7% versus 25.4%, *p* < 0.0001).

### Costs associated with hospitalizations

Although the highest total admission costs were observed among admissions due to chronic organ damage ($ 2176.40 per admission) and VOC ($ 1436.40 per admission), these costs were not significantly different (*p* = 0.106, Fig. 2B). The two highest total costs per admission were observed in two patients (32 and 34 years-old) with HbSS, both hospitalized for VOC, for a total length of stay of 130 and 80 days, and with cost per admission $46,166.35 and $30,157.95, respectively. Both developed ACS, septic shock, multiple end-organ insufficiencies, and failure, including stroke, seizures, and renal failure necessitating dialysis. Among patients with > 3 hospitalizations during the study period, median costs were significantly higher than those with ≤3 hospitalizations ($ 12,444.00 versus US $ 1952.13 per patient, *p* = 0.0001).

Overall, there were no cost differences according to sex, even when stratifying by the different discharge diagnoses (Supplemental Fig. 3F). However, there were differences in cost according to age group, where overall hospitalizations costs among children were higher compared with that of adults ($1886.81 versus $1235.37 per admission, *p* < 0.0001). This difference in cost according to age was also true for the subset of patients with Hb SS or HbSβ^0^-thalassemia, in which median hospitalization costs among children were higher than those for adults ($1885.37 versus $1369.55 per hospitalization, respectively, *p* = 0.0008). (Supplemental Fig. 3E).

Although adults experienced more admissions for VOC than children [190 (61.1%) versus 121 (38.9%), respectively), the highest median cost per VOC admission event was higher among children ($1909.32), compared to that of adults ($1230.45, *p* < 0.0001). An exception related to higher costs of pediatric hospitalizations was infection, however. Although more children experienced hospitalizations for infection (136 events, 82.9% of children) relative to adults (28 events, 17.1% of adults), the median cost for admission among adults ($2529.44) was higher compared to that among children ($1924.18, *p* = 0.0381) (Supplemental Fig. 3G).

Differences in costs were also observed considering sickle genotypes. Costs associated with admission visits for patients with HbSS or HbSβ^0^-thalassemia were higher than those with other genotypes (median $1677.18 vs. $1224.47/visit, *p* < 0.0001) and also among adults with HbSS or HbSβ^0^-thalassemia, compared with those of other sickle genotypes ($1369.55 versus $1024.13, *p* = 0.0031) (Supplemental Fig. 3E).

Finally, costs were higher among those patients who were prescribed hydroxyurea at the time of admission compared with those who were not prescribed this medication (1530.93 versus $1.705.76, *p* = 0.0049, Supplemental Fig. 3H). Results were similar for the subset with HbSS or HbSβ^0^-thalassemia ($1877.93 versus $1577.32, *p* = 0.0099).

### Association between hydroxyurea adherence and acute care costs

Differences in costs were observed relative to hydroxyurea MPR values. To investigate the prognostic value of MPR on acute care costs, we conducted Conditional Inference Trees, [14] a non-parametric method that implements decision tree models through a recursive binary partitioning algorithm. The decision tree algorithm searches for an association between the outcome and a set of covariates. The selection of the explanatory variable is made through an independence test. Variables with significant association at the 10% significance value were selected by the algorithm and displayed in the nodes of the tree with their respective *p*-values. In a regression tree that used admission, sickle genotype and MPR as predictive variables of costs follolwing an ED visits, MPR cutoffs of 52.6, 64.5%, and 24.%, were found to have significant differences in ED costs (Supplemental Fig. 4A: nodes 4 and 5, 7 and 8, and 10 and 11; and 41.7%, respecitively). A second regression tree was built considering admission, reason for ED visit and MPR as predictors of costs following ED visits. In this second tree, the MPR cutoff of 41.7% was found significantly associated with costs (Supplemental Fig. 4B: nodes 4 and 5).

Following identification of the MPR cutoffs, the Wilcoxon rank sum exact test was then used to compare cost differences between each of the 4 pairs of nodes. Among non-admitted patients with HbSS or HbSβ^0^-thalassemia, lower median ED costs were observed among the 40 ED visits with a calculated MPR > 64.5% compared with ED visits with MPR ≤ 64.5% ($98.16 x $182.46, *p* = 0.0007, respectively). Since the number of HbSS or HbSβ^0^-thalassemia ED visits with a MPR ≤64.5% was much greater than 40 (*n* = 1653), a sample of 40 observations among the 1653 entries was extracted matched by sex, age, and reason for the ED visit. The paired Wilcoxon rank sum exact test was performed confirming the lowest cost for the 40 ED visits with MPR > 64.5% (*p* = 0.0043). Lower median ED costs were also seen among non-admitted patients with non-HbSS or HbSβ^0^-thalassemia hemoglobinopathies and with MPR ≤52.6%, relative to those with MPR > 52.6% (median $104.19 x $225.36, respectively *p* = 0.0021). Lower costs were observed among ED visits that resulted in admissions and whose patients had measured hydroxyurea MPR ≤24.4%, compared with those with MPR > 24.45 (median $1743.27 x $2338.35, respectively *p* = 0.0003). Finally, Among ED visits due to anemia exacerbation that did not result in admission, the lower costs corresponded to visits with MPR > 41.7%, compared with those with MPR ≤41.7% (median $274.39 x $387.13, *p* = 0.0064, respectively).

### Combined costs associated with ED and hospitalizations

**Fig. 3:**
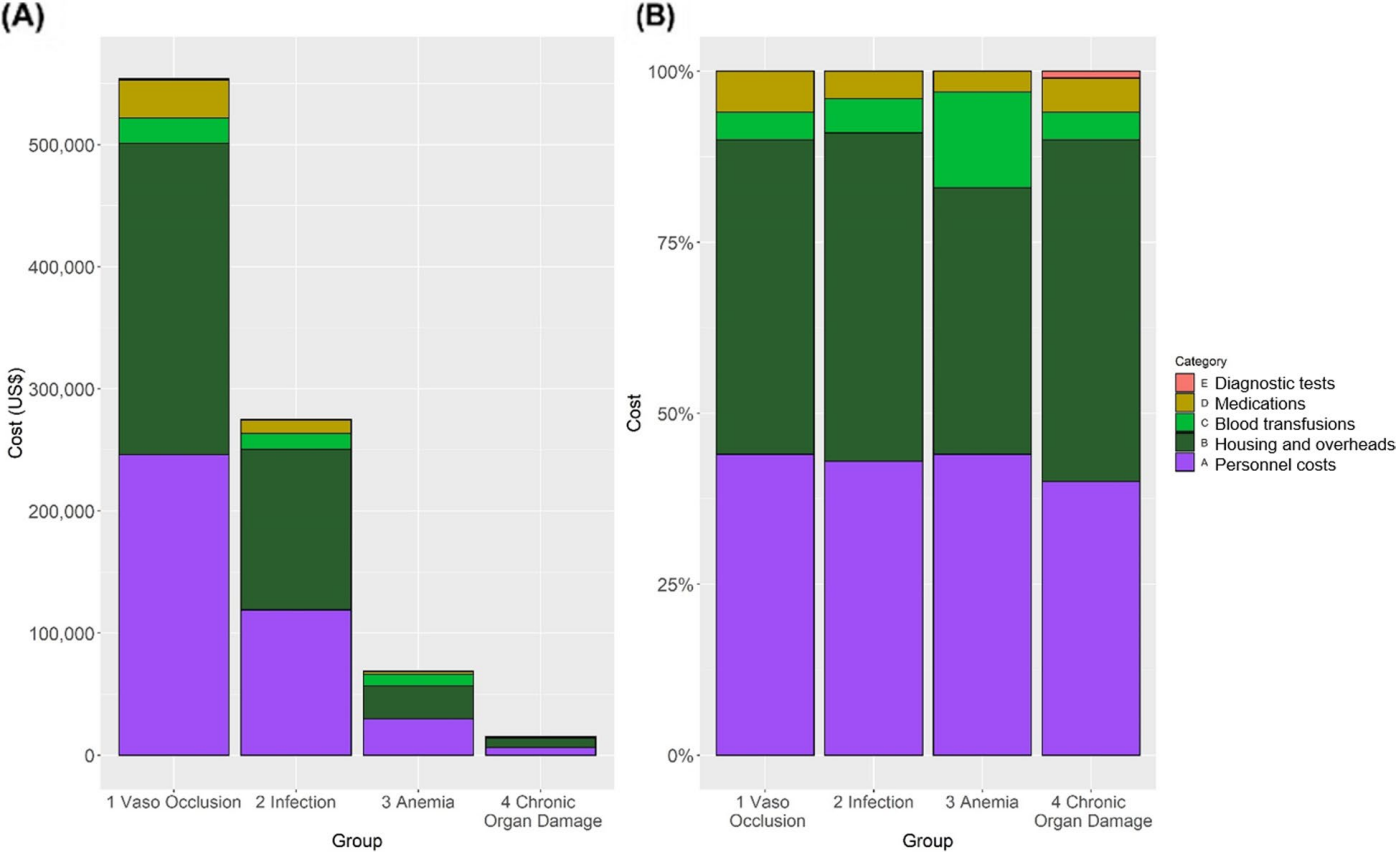
All acute care resource utilization costs. Total Absolute (**A**) and relative (**B**) costs stratified by type of discharge diagnosis group are shown for the costs of emergency department and hospitalization visits. Housing and personnel costs were the highest absolute and relative costs among all acute care visits. The relative blood transfusion costs are highest within the anemia exacerbation group, and medications amount for the third highest relative costs in all four discharge diagnosis groups

**Fig. 4:**
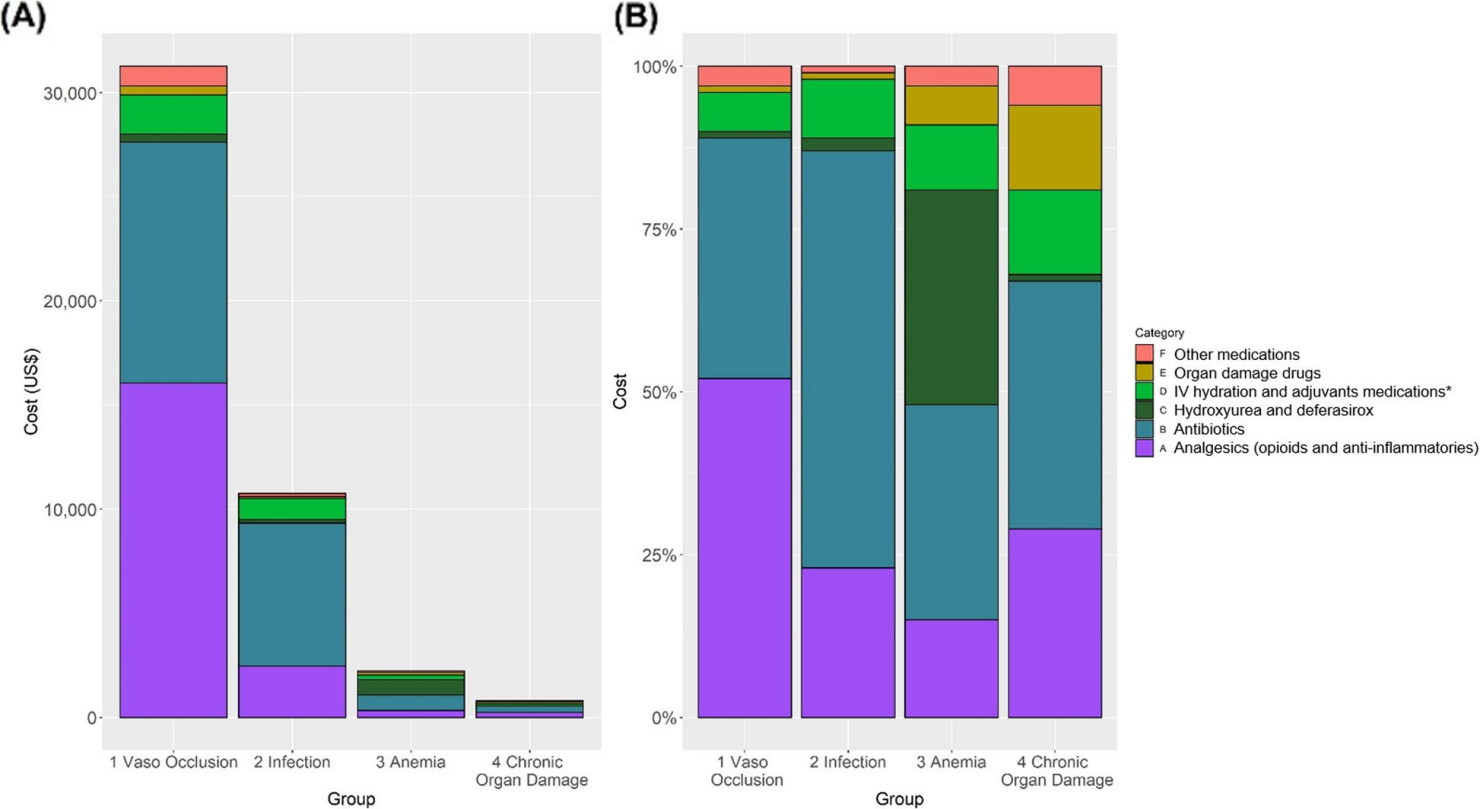
Acute care utilization costs for medications. Absolute (**A**) and relative (**B**) costs for all medications by discharge diagnosis group is shown. Analgesics and antibbiotics together accounted for most of the absolute and relative costs in all diagnoses. The main medication costs drivers in all combined acute care visits for vaso-occlusive pain were analgesic and anti-inflammatory drugs. *Adjuvants include anti-anxiolytics, folic acid, anti-emetics, anti-allergy medications, erythropoietin, sodium chloride, and sodium bicarbonate

ED and admission costs were collapsed to represent the overall costs of all-acute care resource utilization. The absolute and relative costs by variable cost category within each of the discharge diagnostic groups were computed using a microcosting approach (Figs. 3 and 4). Housing and personnel costs accounted for 90% of the absolute cost within each admission group, followed by medications and diagnostic tests (Fig. 3A). Similarly, the relative cost of each microscosting category again showed that housing and personnel contributed to most acute care costs (Fig. 3B), reflecting the impact of the length of stay cost. In analysis of medications alone, analgesics and antibiotics together accounted for 85% of the absolute costs in all discharge diagnoses (Fig. 4A). The main medication cost drivers in all combined acute care visits for VOC were analgesic and anti-inflammatory drugs, representing 52% of the relative costs (Fig. 4B). Among all-acute care visits for infection, 64% of the total relative costs were spent on antibiotics (Fig. 4B). There were no differences in costs of combined ED and admissions relative to hydroxyurea prescribing.

## Discussion

It is estimated that between 60,000 to 100,000 individuals live with SCD in Brazil today [3, 15]. In other LMICs the SCD population is also showing improvements in survival, [16] highlighting the need for better understanding of the costs and resources needed to support continued survival improvements. Clinical guidelines outlining healthcare management of the SCD population specifies early diagnosis, ongoing monitoring, and disease-specific therapies. The optimal delivery of care for hemoglobin disorders, therefore, requires access to highly specialized care centers. As in other LMICs, detailed cost analyses of treatment of SCD complications are not available, precluding appropriate planning of allocation of funds to treatment centers and development of health policies. Further complicating clinical efforts, novel therapeutics to address SCD burden are beginning to enter the marketplace in HIC. Without baseline data on costs, economic evaluation and comparative effectiveness studies to justify LMIC implementation of novel therapeutics are not feasible, thus perpetuating global gaps in outcomes and health disparities.

In our analysis of a single tertiary center in Brazil, over a period of 6 months, approximately one-third of all patients with SCD (52% of children and 48% of adults) at this institution utilized the ED for an acute visit and 33.3% of them were hospitalized for SCD-related acute complications. Although acute care costs per each event for anemia exacerbation were highest among all causes of hospital visits, anemia was a less frequent cause for ED visit or admission, thus, when viewed from the broader health system perspective, VOC was the largest contributor to costs overall. To our knowledge, this is the first report of costs associated to acute care treatment of patients with SCD in Brazil.

The multiple complications seen in SCD contribute to significant morbidity and premature mortality, as well as substantial costs to the healthcare system [17]. Chronic end-organ damage can significantly increase the costs associated with the treatment in SCD and in our analysis, acute complications of end-organ damage represented the highest costs during hospitalizations. Although conducted in a LMIC country, our results correlate and are corroborated by published data among adults in the United States insured under government Medicaid plans showing high costs associated with end-organ damage [18]. Furthermore, our microcosting analysis reveals that pharmacy costs, housing, and personnel time accounted for most of the cost related to ED and inpatient visits in our analysis. These findings are also in accord with data from SCD patients insured under the Medicaid government plan in the United States, which showed pharmacy and inpatient costs to be main drivers of high costs in SCD [19].

Identifying opportunities for cost-savings is a critical secondary output from our analysis. Prior cost-savings with hydroxyurea therapy have been shown in children who participated in the BABYHUG clinical trial study [20]. However, few studies have investigated the cost-savings of disease-modifying therapies in SCD in real-world settings. Our results suggest a lower acute resource utilization with hydroxyurea, as acute care visits by treated patients were less common than that by untreated ones. Among individuals with higher medication adherence levels, cost-savings with hydroxyurea therapy were also suggested by a reduction in costs, but only among visits by patients with the most severe sickle genotypes (HbSS or HbSβ^0^-thalassemia) who were not admitted following an ED visit and during ED visits for anemia exacerbation. Our findings should be interpreted with caution, as adherence was overall low (well below the desired goal of 80%) and our analysis excluded treated patients not seen in the ED during the study period. Our analysis failed to show cost reductions with hydroxyurea during admissions, likely reflecting the limits of a disease-modifying therapy in reducing costs in the face of greater disease severity, where costs are inherently higher. Our findings, however, underscore the importance of interventions to improve hydroxyurea prescribing by providers and adherence by patients. To better understand the potential role of hydroxyurea in reducing care costs in SCD, our future work will investigate the relationship between hydroxyurea adherence and utilization for the entire SCD population (with and without ED utilization) of HEMORIO and will include not only acute care costs, but also ambulatory costs.

Although not included in this analysis, new disease-modifying therapies that can reduce acute SCD complications (crizanlizumab [21] and L-glutamine [22]) and reduce anemia (voxelotor [23]) have recently transitioned to standard of care in HICs and will soon become available in LMICs. As to our knowledge, no cost-benefit or cost-effectiveness analyses have been done based on these new drugs, our study will serve as a critical benchmark against which these new therapies may be compared. Our findings can, for instance, be used in cost-effectiveness analyses to inform policy decisions regarding coverage of new disease-modifying drugs in LMICs, based on their estimated cost-savings potential during acute events from SCD.

Costs associated with admissions for acute VOC among children were generally higher than for adults. Although the reasons are unclear, longer duration of admissions (approximately twice as long) and higher use of blood products among children, as compared with adults, potentially explains these differences. In a retrospective cost analysis in England, children ages 10 to 19 years were also more likely to have longer inpatient lengths of stay compared to other age groups [24]. Care utilization differences among adults and children in Brazil warrant future evaluations of how implementation of evidence-based practices occur in LMICs among adults.

The two highest hospitalization costs in our analysis were observed in two patients with HbSS who developed multiple complications following an admission for vaso-occlusive pain (sepsis and multi-organ failure), substantially prolonging their lengths of stay. Patients with high frequency of hospitalizations (high utilizers) also incurred in high utilization costs. Further, our overall LOS was high (mean of 2.8 days for adults) contrasting with a study from England that showed that more than half of the adults were discharged within 24 h, [25] suggesting opportunities to enhance ambulatory preventative services. Collectively, our results highlight a critical need to develop new tools to identify high-risk groups of patients with SCD as a strategy to mitigate the cost burden of SCD impact on the health system.

Although presenting important cost information, our study had limitations. First, we only included data from one center in Brazil, limiting generalizability. However, our data include real costs from vendors and distributors. While practice differences may vary by location, our findings are likely similar to those observed at other Brazilian institutions. Second, our analysis was limited to 6 months of observation but given our large population and no seasonal changes from prior and subsequent months, our data are likely representative of longer periods and different times of the year. The strength of our study lies in its scope in that it covers the most common diagnoses of acute complications from SCD in the ED and inpatient settings and provides a comprehensive description of acute care costs with a detailed account of different types of medical expenses. Additionally, our cost data were not estimated from existing schedule of payment sources or extrapolated from other sources, but rather, used real-world cost data.

In conclusion, our study showed that SCD incurs in high costs in a tertiary hematology LMIC center, both during ED visits and inpatient stays, especially for the treatment of anemia and chronic end-organ damage. Drivers of cost included housing and personnel. Hospitalization costs were higher among children compared with adults. Hydroxyurea therapy appears to reduce costs, but only for ED visits among patients with HbSS/HbSβ^0^-thalassemia and anemia exacerbation, and was dependent on adherence level. Hydroxyurea use should be expanded to both improve patient outcomes and potentially produce future cost-saving to the health care system by reducing acute care utilization and ED costs. Our data provide benchmarks to be used in future cost-effectiveness analysis to plan new care guidelines and estimate cost benefits of current and future therapies for SCD.

## Supplementary Information

**Additional file 1: Supplemental Table 1.** Standars of care for the treatment of acute complications of sickle cell disease in Brazil according to the Brazilian Ministry of Health (PROTOCOLO CLÍNICO E DIRETRIZES TERAPÊUTICAS PARA DOENÇA FALCIFORME (conitec.gov.br).

**Additional file 2: Supplemental Table 2**. Monthly costs of services by type of visit.

**Additional file 3: Supplemental Table 3.** Diagnoses of ED visits excluded from analysis.

**Additional file 4: Suplementary Figure 1**.

**Additional file 5: Suplementary Figure 2**.

**Additional file 6: Suplementary Figure 3**.

**Additional file 7: Suplementary Figure 4**.

## Data Availability

The datasets used and/or analyzed during the current study are available from the corresponding author on reasonable request.

## Abbreviations

SCD: Sickle cell disease
ED: Emergency department
HU: Hydroxyurea
VOC: Vaso-Occlusion
PRBC: Packed red blood cell
MPR: Medication Possession Ratio
HIC: High- income countries
LMIC: Low- and middle-income countries
