# Supplementary material for: Cost analysis of acute care resource utilization among individuals with sickle cell disease in a middle-income country
Source: BMC Health Serv Res. 2022 Jan 8;22:42. doi: 10.1186/s12913-021-07461-6 (PMC8742916; doi:10.1186/s12913-021-07461-6)
Supplement: Supplementary file 1 — Additional file 1: Supplemental Table 1. Standars of care for the treatment of acute complications of sickle cell disease in Brazil according to the Brazilian Ministry of Health (PROTOCOLO CLÍNICO E DIRETRIZES TERAPÊUTICAS PARA DOENÇA FALCIFORME (conitec.gov.br). [file 12913_2021_7461_MOESM1_ESM.docx]

**Supplemental Table 1.** Standars of care for the treatment of acute complications of sickle cell disease in Brazil according to the Brazilian Ministry of Health ([PROTOCOLO CLÍNICO E DIRETRIZES TERAPÊUTICAS PARA DOENÇA FALCIFORME (conitec.gov.br)](http://conitec.gov.br/images/Protocolos/PCDT_DoencaFalciforme_2018.pdf).

| Reason for acute hospital visit | Treatment according to national standards |
| --- | --- |
| Vaso-occlusive pain | Intravenous non-opioid analgesics, such as dipyrone (pyrazalone derivative), non-steroidal anti-inflammatories, and opioids (codeine, methadone, or morphine).  During pain management, the patient with SCD is monitored with pulse oximetry (hemoglobin oxygen saturation) and chest X-rays performed if chest pain or worsening of blood gas analysis.  In case of symptoms of worsened anemia or a drop of 2 g/dL in the baseline hemoglobin level without compensatory reticulocytosis, a simple red blood cell transfusion is given. |
| Acute chest syndrome | Avoidance of over-hydration. Respiratory therapy, and intravenous antibiotics, such as cefuroxime, azithromycin and ofloxacin are used.  The treatment of acute anemia is done with simple transfusion of leuko-depleted red blood cell units that are minor-antigen matched. |
| Fever (presumed sepsis) | Laboratory tests (complete blood count, reticulocyte count), blood culture and urine cultures when applicable, intravenous hydration and broad-spectrum antibiotics such as cefuroxime and azithromycin for 48 until final cultures result. |
| Chronic end-organ dysfunction/failure | Disease-modifying therapies (e.g., chronic transfusions and hydroxyurea), in addition to treatments that are organ-specific, such as calcium channel inhibitors or hemodialysis for chronic kidney dysfunction and renal failure, respectively.  Imaging studies if indicated (e.g., chest X-ray if respiratory symptoms) and laboratory tests, including complete blood count, are done during emergency department and hospitalization visits, as indicated. |
