# Supplementary material for: Cost analysis of acute care resource utilization among individuals with sickle cell disease in a middle-income country
Source: BMC Health Serv Res. 2022 Jan 8;22:42. doi: 10.1186/s12913-021-07461-6 (PMC8742916; doi:10.1186/s12913-021-07461-6)
Supplement: Supplementary file 2 — Additional file 2: Supplemental Table 2. Monthly costs of services by type of visit. [file 12913_2021_7461_MOESM2_ESM.docx]

**Supplemental Table 2: Monthly costs of services by type of visit.**

Notes: ED= emergency department, ADM = admission
