# Supplementary material for: Cost analysis of acute care resource utilization among individuals with sickle cell disease in a middle-income country
Source: BMC Health Serv Res. 2022 Jan 8;22:42. doi: 10.1186/s12913-021-07461-6 (PMC8742916; doi:10.1186/s12913-021-07461-6)
Supplement: Supplementary file 3 — Additional file 3: Supplemental Table 3. Diagnoses of ED visits excluded from analysis. [file 12913_2021_7461_MOESM3_ESM.docx]

**Supplemental Table 3.** Diagnoses of ED visits excluded from analysis

| Cause of exclusion | Number of visits |
| --- | --- |
| Allergic rhinitis | 2 |
| Analgesia for dresssing change | 1 |
| Anxiety | 4 |
| Bell's paralysis | 1 |
| Outpatient imaging test | 1 |
| Hydroxyurea management discussion | 1 |
| Left against medical advice (diagnosis not made) | 2 |
| Medication refill request | 2 |
| Gastroenteritis | 2 |
| Non-acute labwork | 5 |
| Scheduled monthly transfusion | 3 |
| Trauma | 1 |
| Vaccine indication discussion | 4 |
| Total | **29** |
