# Supplementary figures and images for: Cost analysis of acute care resource utilization among individuals with sickle cell disease in a middle-income country
Source: BMC Health Serv Res. 2022 Jan 8;22:42. doi: 10.1186/s12913-021-07461-6 (PMC8742916; doi:10.1186/s12913-021-07461-6)

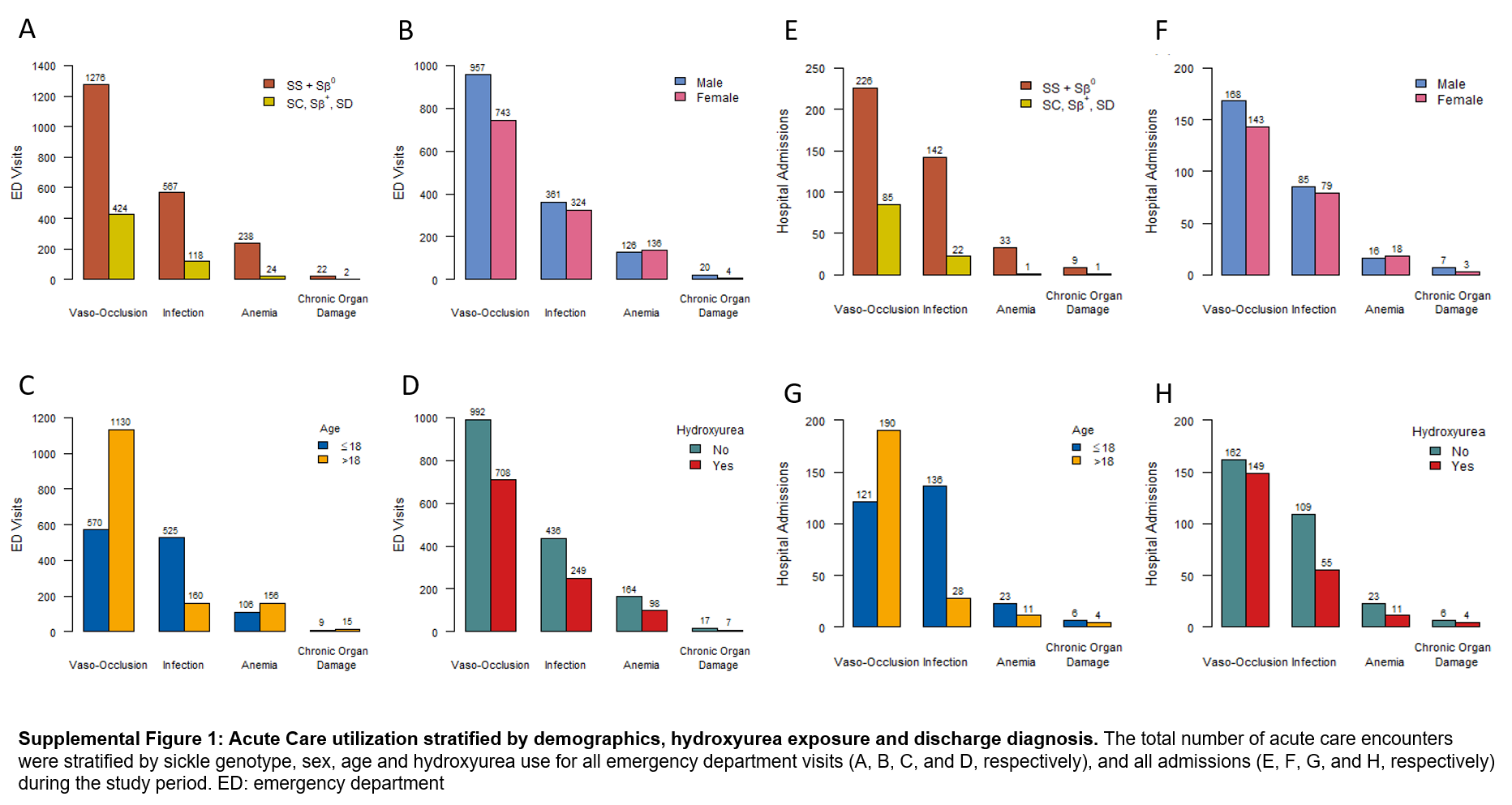

Supplement: Supplementary file 4 — Additional file 4: Suplementary Figure 1. [file 12913_2021_7461_MOESM4_ESM.png]

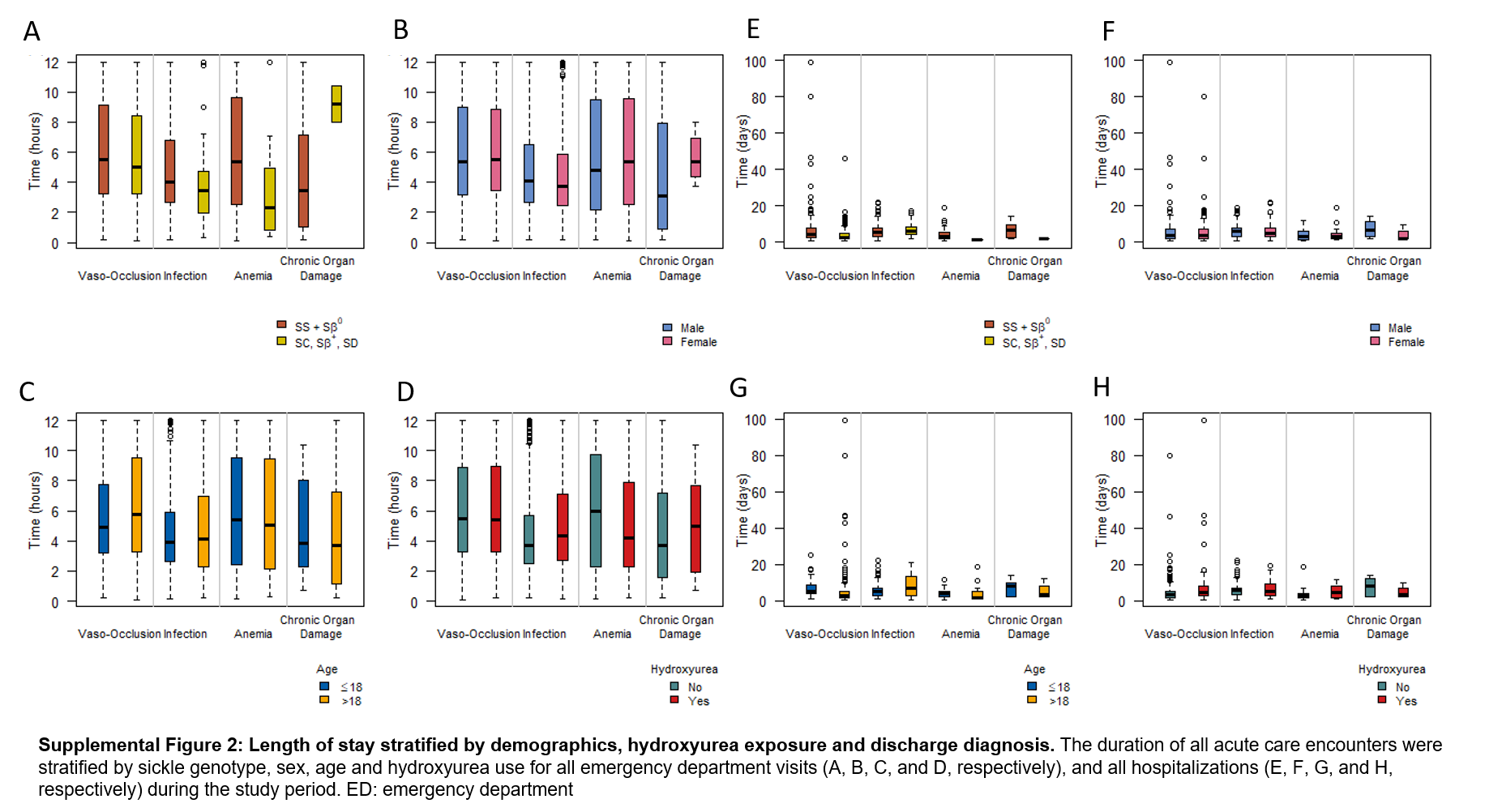

Supplement: Supplementary file 5 — Additional file 5: Suplementary Figure 2. [file 12913_2021_7461_MOESM5_ESM.png]

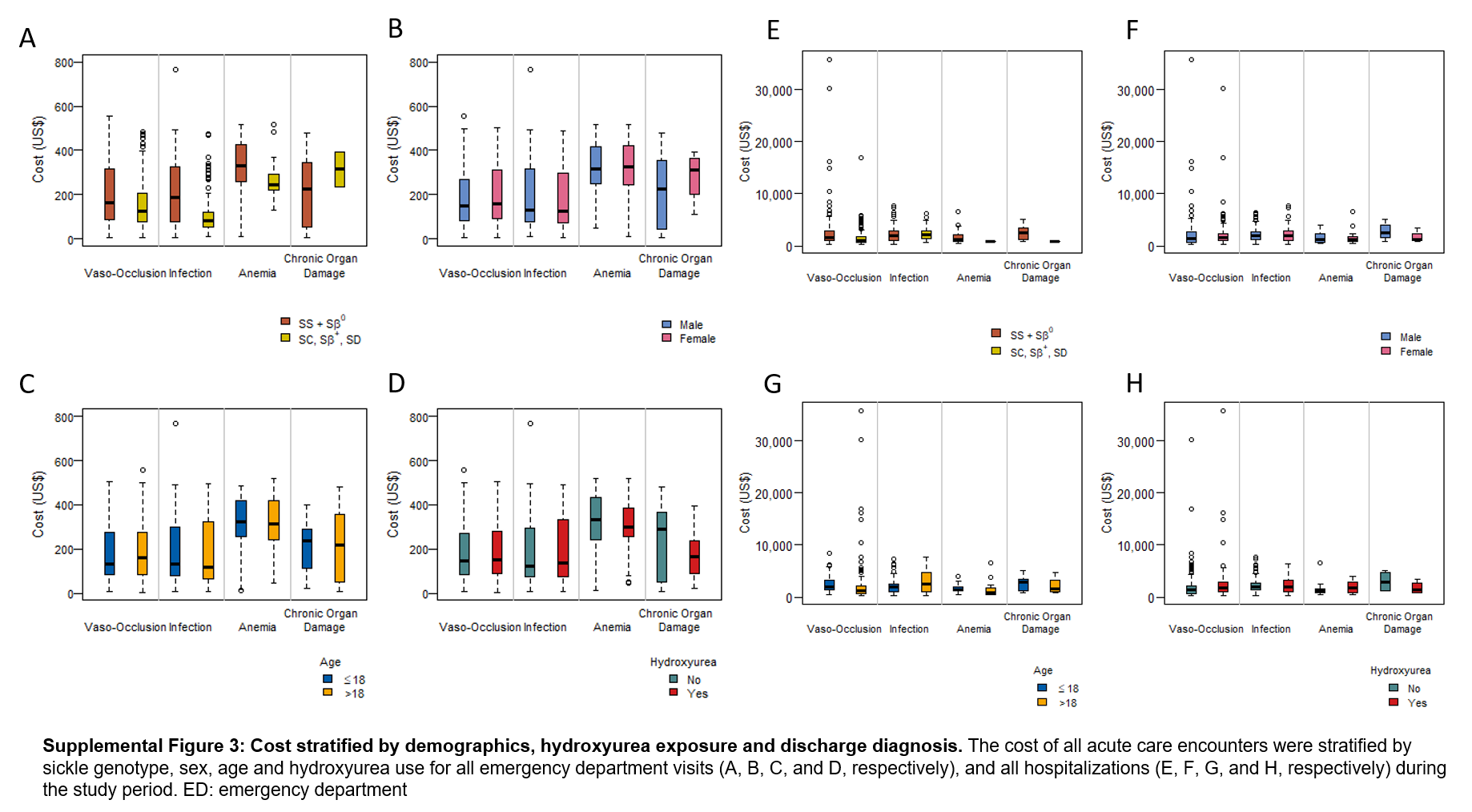

Supplement: Supplementary file 6 — Additional file 6: Suplementary Figure 3. [file 12913_2021_7461_MOESM6_ESM.png]

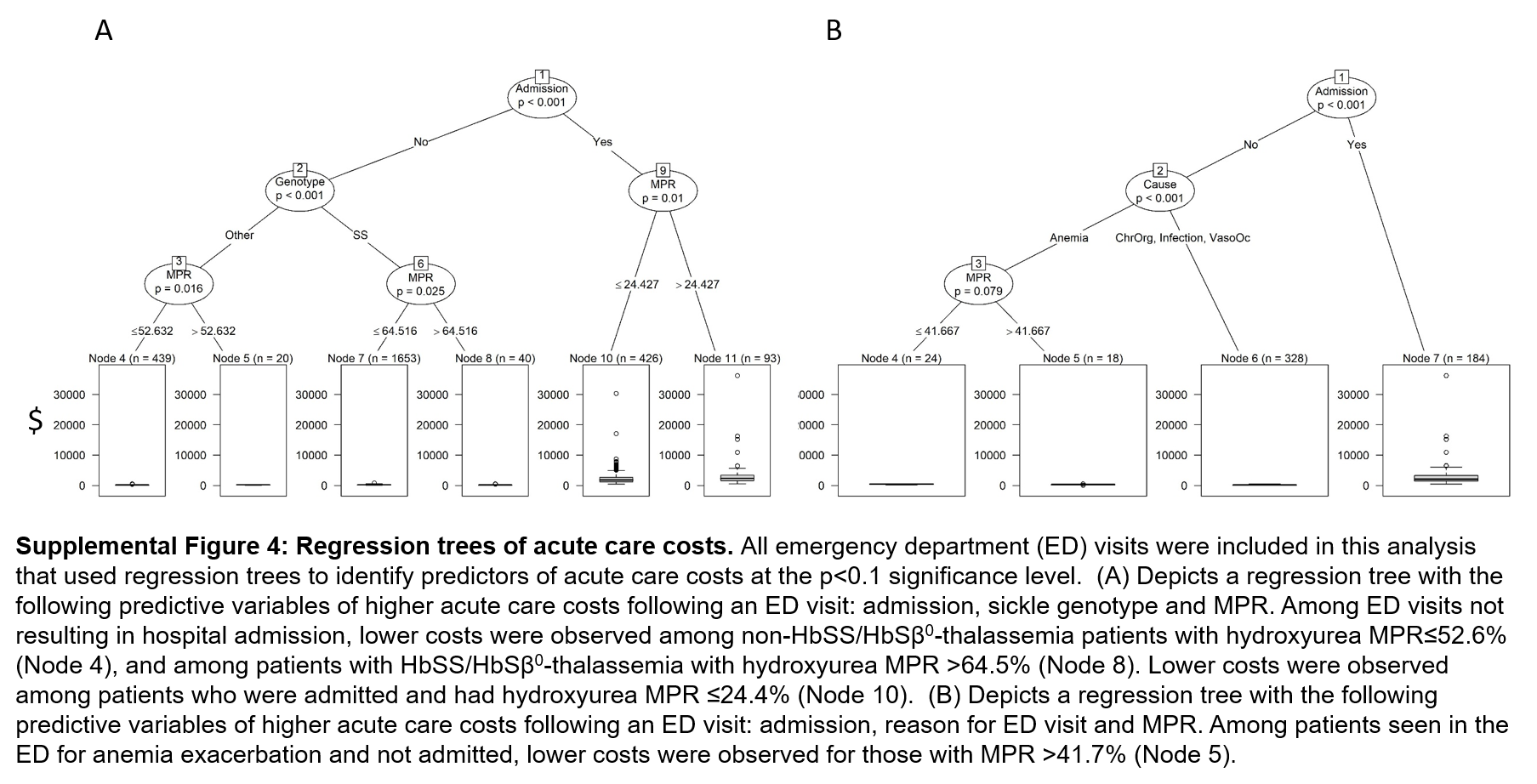

Supplement: Supplementary file 7 — Additional file 7: Suplementary Figure 4. [file 12913_2021_7461_MOESM7_ESM.png]
